# Anterior Perineum: Between the Gynecological and Urinary Tract

**DOI:** 10.5334/jbsr.3113

**Published:** 2023-04-11

**Authors:** Guillaume Puissant, Cristina Anca Dragean, Isabelle Leconte

**Affiliations:** 1 Cliniques Universitaires Saint-Luc, BE

**Keywords:** Urethral diverticulum, pelvis, MRI

## Abstract

**Teaching Point:** Female urethral diverticulum is a rare condition that is often a diagnostic challenge; magnetic resonance imaging (MRI) is efficient to confirm the diagnosis (especially if endovaginal ultrasound is inconclusive), to assess the diverticulum prior to surgery, and to detect related complications including intra-diverticular neoplasm.

## Case History

A 76-year-old female patient presented to the emergency room for persistent metrorrhagia. Gynecological examination revealed erosions of the anterior vaginal mucosae with an underlying palpable para-vaginal mass.

**Figure 1:**
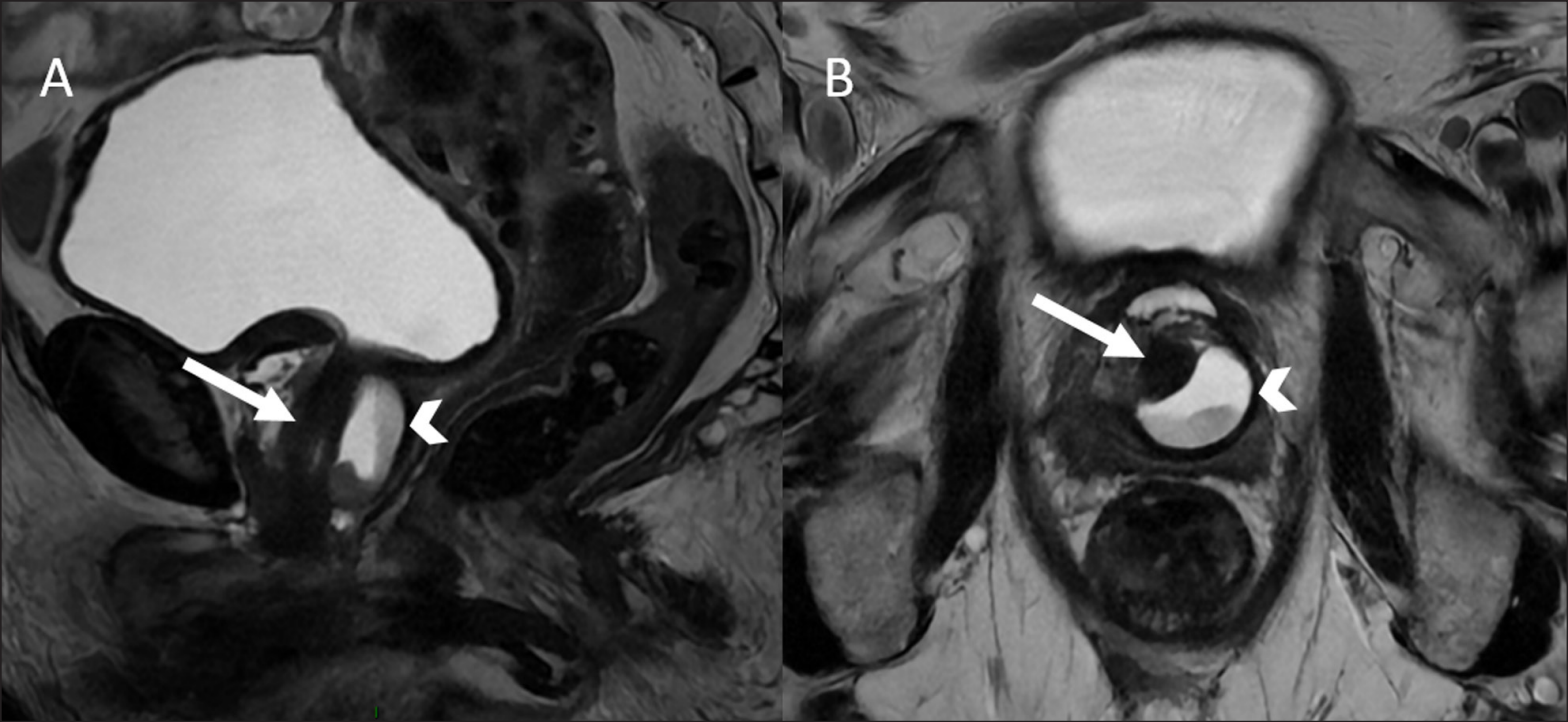


An MRI was performed to assess this mass with T2-weighted images in coronal, sagittal, and axial planes, DWI (bo,b1000) and T1-weighted fat-suppressed images with and without contrast injection. T2-weighted images in sagittal (Figure 1A) and axial (Figure 1B) plane revealed a high signal intensity lesion with a fluid level (Figure 1A and B, arrowhead) surrounding the urethra (Figure 1A and B, white arrow) corresponding to a urethral diverticulum.

**Figure 2:**
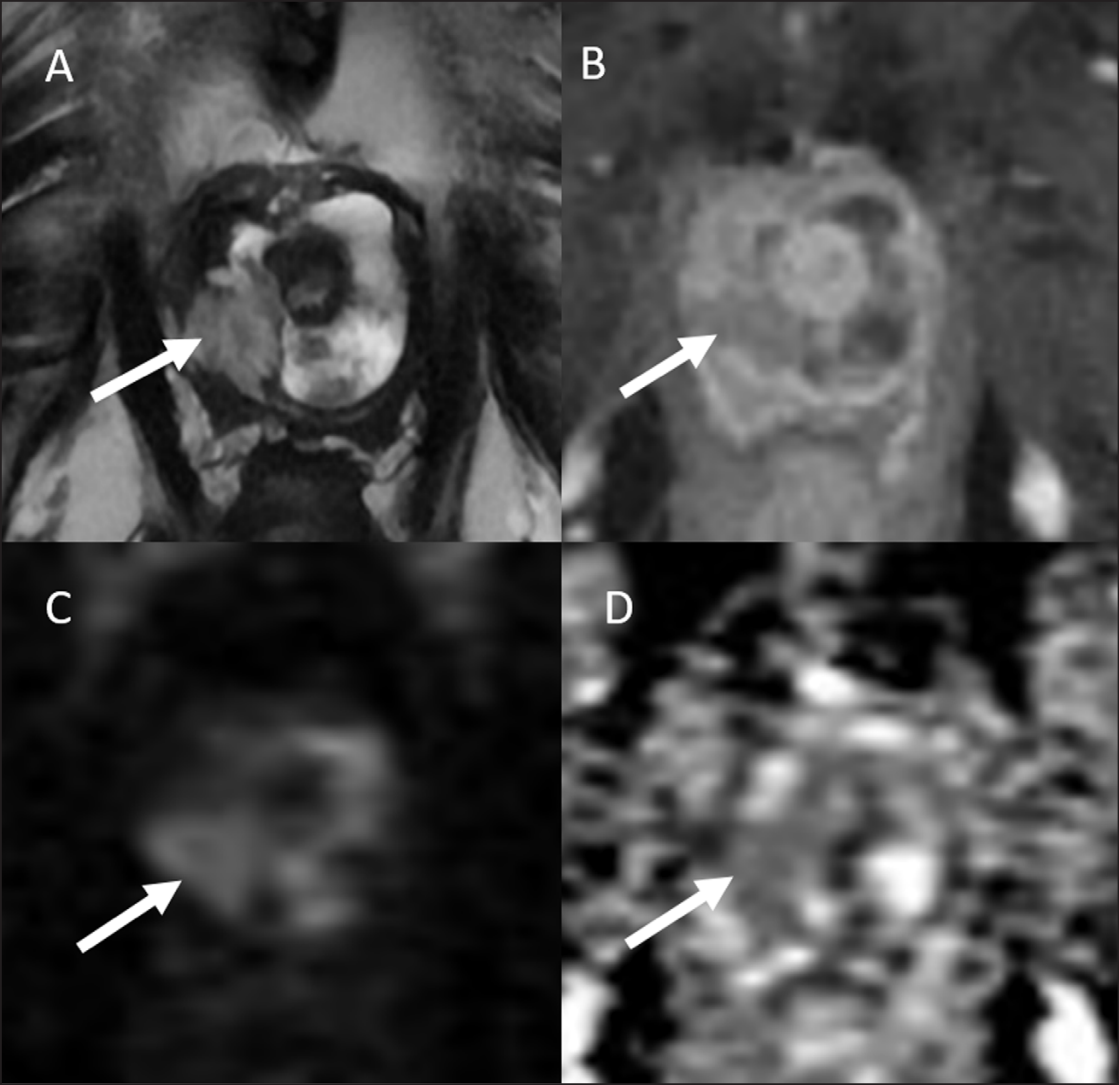


MRI also showed a solid part on the right posterolateral side of the diverticulum (Figure 2) characterized by a T2 slight hypersignal on T2-weighted images (Figure 2A, white arrow), enhancement on axial T1-weighted fat-suppressed images (Figure 2B, white arrow) with marked restriction diffusion (Figure 2C, white arrow) and low ADC value (1.051 × 10^–6^ s/mm^2^) (Figure 2D, white arrow). These characteristics are highly suspicious of intra-diverticular malignant transformation.

**Figure 3:**
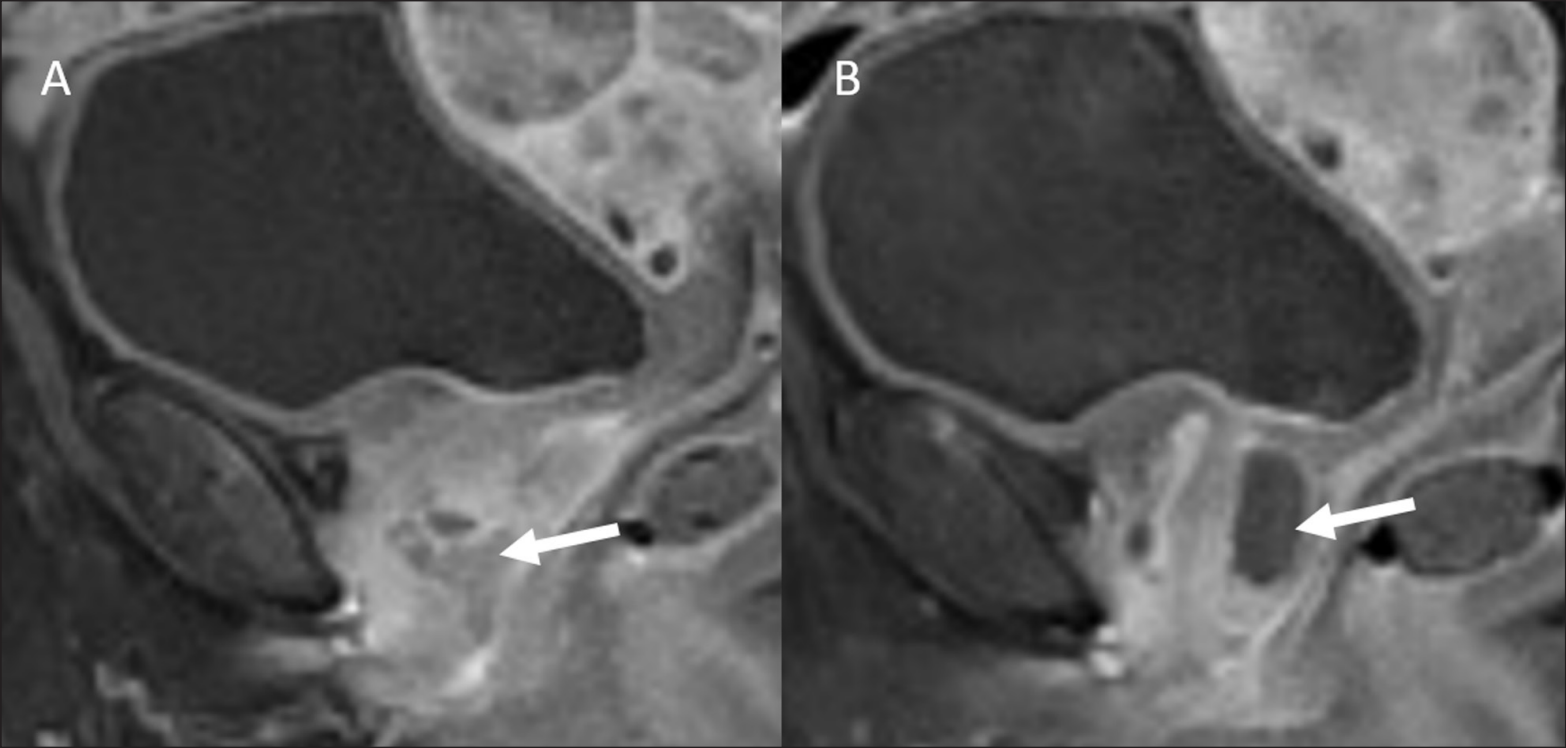


The enhancement of the intra-diverticular neoplasm is also well seen on sagittal T1-weighted fat-suppressed images (Figure 3) with a significant enhancement difference between the malignant portion (Figure 3A, white arrow) and the cystic portion (Figure 3B, white arrow) of the urethral diverticulum (Figure 3B, white arrow).

The patient underwent anterior pelvectomy. Anatomopathology confirmed the malignancy and the diagnosis of clear cell carcinoma.

## Comments

Female urethral diverticulum is a rare condition affecting 0.6 to 6% of women, mostly between the third and sixth decades. Clinical presentation includes a large variety of urologic symptoms (repeated lower infections, dysuria, dyspareunia, and post-voiding dribbling), and the diagnosis remains a diagnostic challenge [1].

Urethral diverticula are located between the fibromuscular layer of the urethra and the anterior wall of the vagina. Endovaginal ultrasound is an effective first-line exam to confirm or exclude the diagnosis, especially in younger women. Because of its soft tissue contrast, MRI is accurate to assess the urethral diverticulum’s morphology before surgery and to evaluate its internal content [1].

The location of the cystic lesion in the postero-lateral side of the mid-urethra, and the evidence of communication between the cyst and the urethral lumen are the key features to differentiate urethral diverticulum from vaginal cysts. Urethral diverticula may also have horse-shoe configuration that can occasionally almost completely surround the urethra [1].

MRI also allows assessment of the possible complications of urethral diverticulum including infection, calculi formation, and intra-diverticular neoplasm that usually appears as a gadolinium enhanced mass in the diverticulum [1].
